# Interindividual variability of electric fields during transcranial temporal interference stimulation (tTIS)

**DOI:** 10.1038/s41598-021-99749-0

**Published:** 2021-10-13

**Authors:** Jill von Conta, Florian H. Kasten, Branislava Ćurčić-Blake, André Aleman, Axel Thielscher, Christoph S. Herrmann

**Affiliations:** 1 Experimental Psychology Lab, Department of Psychology, European Medical School, Cluster of Excellence “Hearing4All”, Carl Von Ossietzky University, Ammerländer Heerstr. 114–118, 26129 Oldenburg, Germany; 2 Neuroimaging Unit, European Medical School, Carl Von Ossietzky University, Oldenburg, Germany; 3 Department of Biomedical Sciences of Cells and Systems, University of Groningen, University Medical Center Groningen, Groningen, The Netherlands; 4 Danish Research Centre for Magnetic Resonance, Center for Functional and Diagnostic Imaging and Research, Copenhagen University Hospital Amager and Hvidovre, Copenhagen, Denmark; 5 Center for Magnetic Resonance, DTU Health Tech, Technical University of Denmark, Kgs Lyngby, Denmark; 6 Research Center Neurosensory Science, Carl Von Ossietzky University, Oldenburg, Germany

**Keywords:** Neurophysiology, Neuroscience

## Abstract

Transcranial temporal interference stimulation (tTIS) is a novel non-invasive brain stimulation technique for electrical stimulation of neurons at depth. Deep brain regions are generally small in size, making precise targeting a necessity. The variability of electric fields across individual subjects resulting from the same tTIS montages is unknown so far and may be of major concern for precise tTIS targeting. Therefore, the aim of the current study is to investigate the variability of the electric fields due to tTIS across 25 subjects. To this end, the electric fields of different electrode montages consisting of two electrode pairs with different center frequencies were simulated in order to target selected regions-of-interest (ROIs) with tTIS. Moreover, we set out to compare the electric fields of tTIS with the electric fields of conventional tACS. The latter were also based on two electrode pairs, which, however, were driven in phase at a common frequency. Our results showed that the electric field strengths inside the ROIs (left hippocampus, left motor area and thalamus) during tTIS are variable on single subject level. In addition, tTIS stimulates more focally as compared to tACS with much weaker co-stimulation of cortical areas close to the stimulation electrodes. Electric fields inside the ROI were, however, comparable for both methods. Overall, our results emphasize the potential benefits of tTIS for the stimulation of deep targets, over conventional tACS. However, they also indicate a need for individualized stimulation montages to leverage the method to its fullest potential.

## Introduction

Electric brain stimulation techniques, such as transcranial alternating current stimulation (tACS), are widely used to modulate human brain activity on a cortical level^1–3^. It is, however, not possible to reach deep brain structures non-invasively without stimulating the overlaying cortex. Currently, it is solely possible to target deep brain structures focally with an invasive brain stimulation technique, namely deep brain stimulation (DBS). DBS is used as a powerful therapeutical technique for several clinical disorders such as Parkinson’s disease^4–6^, treatment-resistant depression^7–12^, or Alzheimer’s disease^13–15^. However, DBS is based on a different mechanism of action (supra-threshold activation) as compared to non-invasive electric brain stimulation techniques, like tACS (sub-threshold modulation).

**Figure 1:**
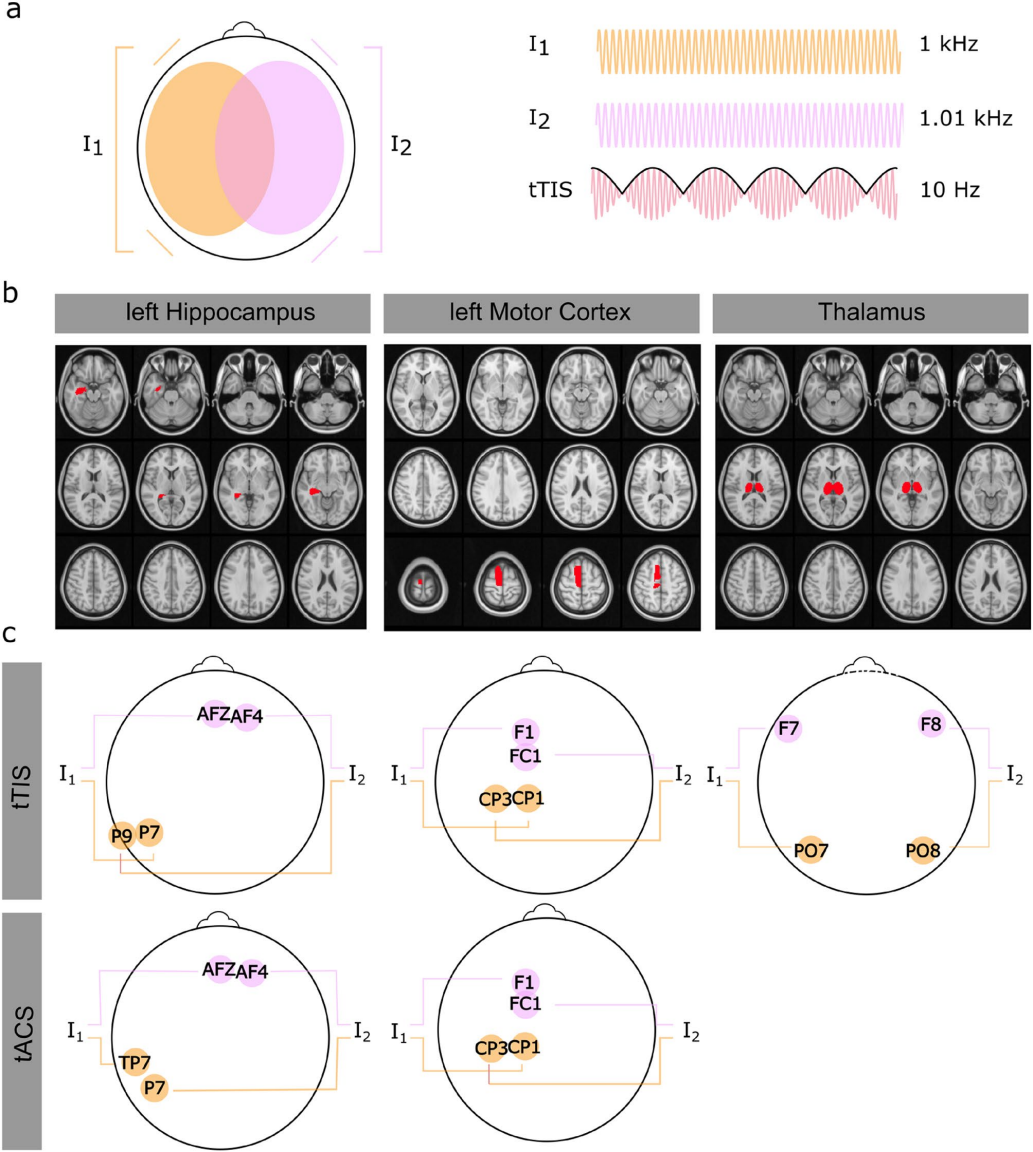
Concept of temporal interference stimulation (tTIS) and selected electrode montages to target three different regions of interest (ROIs). (**a**) For tTIS, two pairs of stimulation electrodes are attached to the scalp. Between the electrodes of each electrode pair, a high frequency alternating current is applied at e.g., 1 kHz and 1.01 kHz. The superposition of the signals causes an amplitude modulation oscillating at the difference of both frequencies (in this case 10 Hz). (**b**) Anatomical locations (in red) of the defined ROIs on the MNI brain: left hippocampus, left motor cortex and thalamus. (**c**) The electrode montages targeting the left hippocampus and the left motor cortex were adapted from Rampersad et al.^26^ for tTIS and for tACS. The electrode montage targeting the thalamus was defined with F7-PO7, and F8-PO8 for tTIS and for tACS.

Based on similar mechanism of action as tACS, transcranial temporal interference stimulation (tTIS) has been developed as a new non-invasive brain stimulation technique to electrically stimulate neurons at depth^16^. For tTIS, electric currents are applied through multiple electrode pairs at two different high frequencies (f_1_, f_2_, with f_1_ < f_2_) that are not in the range of regular neural frequencies (i.e., > 600 Hz) but they do penetrate the skull and brain tissue^17^. The applied currents stimulate with slightly different frequencies. The beat frequency of these two currents oscillates at f_diff_ (f_diff_ = f_2_ − f_1_), which represents the target stimulation frequency. This beat frequency is maximal when the two signals overlap with same strength and the field vectors have the same direction (Fig. 1a). It was demonstrated that tTIS triggers neural firing in the hippocampus of mice at f_diff_^16^. Moreover, it has been shown that neurons were activated only in deep brain structures (where both stimulation frequencies overlap with same strength), without stimulating the overlaying cortex^16^. Furthermore, it was shown that tTIS over the left motor cortex led to motor activity in mice^16^. However, it could be challenging to translate knowledge gained in animal studies to humans: two-electrode tES is approx. 100-times stronger in the cortex of a mouse than in the human model. Generally, electric field strength due to tES strongly decreases from smaller to larger head sizes^18^. In contrast to mice, the human brain cannot be stimulated with supra-threshold field strength (as the mice in the tTIS study mentioned above^16^).

The efficacy of tTIS to modulate neural oscillations in the human brain has not been investigated so far and current research is limited to animal studies, computational models and electric field simulations. The putative neurobiological mechanisms of tTIS has been investigated^19^ using a single neuron computational model. In this model, classical Hodgkin–Huxley squid neurons and some mammalian cells exhibited effects during temporal interference stimulation. However, some of the mammalian cells in the study did not show an effect to temporal interference stimulation. Another study showed that pure high frequency sinusoids resulted in a current balance between inward and outward current in single neuron models^20^. This current balance restrains the membrane potential from spiking. The fast-changing envelope, resulting when both high frequency sinusoids overlap, activates fast depolarizing currents without giving slow outward currents time to respond. This imbalance causes the neuron to fire (note that this study is based on a single neuron model and field strength achievable with available non-invasive electric brain stimulation techniques are far too week to cause neuron firing in humans). Other research investigated the suprathreshold and subthreshold membrane dynamics of neurons in response to interferential stimulation and the authors showed in a neuron model that tTIS was able to modulate spiking activity and facilitated phase synchronization similar to a relatively well-established brain stimulation technique, namely transcranial alternating current stimulation (tACS)^21^. Compared to conventional tACS, it should be noted though, that the modulatory effects were less potent for tTIS at similar field intensities, which is in line with other simulation work on the effect of electric stimulation with amplitude modulated signals^22^. A study based on hippocampal slices of rats showed that the spatial selectivity of tTIS (superficial brain regions stay unmodulated and only deep brain targets are modulated) depends on phasic modulation of neural oscillations in deep brain structures^23^. Another study suggested that the mechanism of tTIS is based on an ion-channel mediated signal rectification process, instead of passive membrane filtering (as suggested in previous studies)^24^.

In addition to the investigation of the neurobiological mechanisms of tTIS, simulation studies focused on the exploration of the resulting electric fields during tTIS in humans. These showed that the stimulation intensity of tTIS might be similar to conventional transcranial alternating current stimulation (tACS)^25,26^. However, it was also shown that tTIS goes beyond superficial areas and may reach deeper areas^26^, in addition to producing more focal fields than conventional tES methods. A recent study proposed to use optimization algorithms to target desired ROI’s inside the brain when stimulating with tTIS^27^. With such algorithms, the authors showed that tTIS has the potential for focal non-invasive deep brain stimulation. Another study showed that optimal stimulation conditions with temporal interfering fields are inconsistent across subjects and suggested to consider individual anatomical differences^28^. However, the study is based on only three individual subjects and the used optimization algorithm seems to be time-consuming. Therefore, further research is needed to investigate the individual variability of the resulting electrical field during tTIS on a larger population.

A computational study^26^ that aimed to optimize tTIS electrode montages for target field strength and focality investigated how different parameters influence the electric field of tTIS. Therefore, the electrode montages (88 electrode positions) and the stimulation current strength were systematically varied (146 M current patterns). Optimized electrode positions to maximize the electric field with tTIS in three brain regions were developed: the pallidum, the left hippocampus and the left motor cortex. However, optimal electrode placements were analyzed for a single subject, indicating the necessity for simulations across multiple subjects.

In order to optimize stimulation effects of non-invasive electric stimulation techniques, important parameters that have a crucial influence on the effect have to be considered. These include the strength of the electric field and its direction. Stimulation effects are reported for electric field strength inside the human brain of approx. 0.1–0.3 V/m^29–31^. Moreover, it was shown that neurons respond preferentially to stimulation when the field is oriented along the predominant direction of the neuron^32–34^. Therefore, the direction of the applied electric field has also to be considered when applying different brain stimulation techniques. Overall, the localization of the target (where are the neurons located that are related to the expected effect?), the strength of the electrical stimulation (which strength is needed in the target location?), and the preferred direction of the electric field (which direction of the electric field is needed in the target location?) have to be defined in order to achieve optimal stimulation effects with non-invasive electric brain stimulation techniques.

Effects of different non-invasive brain stimulation techniques are controversially discussed in current literature. This discrepancy might be in part due to the variability of electric fields due to different non-invasive brain stimulation techniques^35^, that leads to variable effects across subjects and studies. Due to the different brain anatomies across individuals, it might be beneficial to individually adjust electrode placement in order to target the region of interest (ROI) inside the brain and achieve similar stimulation effects across subjects and studies. In order to target deep brain structures (which require relatively focal stimulation due to their small size), targeting the ROI precisely is inevitable in order to achieve comparable effects. A recent study investigated whether individual differences across subjects regarding the electrical field lead to different stimulation effects for tACS^35^. Since tTIS likely has a similar mechanism of action as tACS and, moreover, the electric fields of tTIS seem to be more focal^26^, it is important to investigate whether the electric fields also differ across subjects with the same electrode montage^26^. Individual differences of the electric fields due to tTIS could lead to variable stimulation effects. However, the variability of electric fields across individual subjects is nearly unknown for tTIS. Therefore, the aim of the current study is to investigate the variability of the electric fields due to tTIS across subjects. Due to similar underlying mechanisms of action, we set out to compare the electrical field of conventional tACS with the electrical field of tTIS.

## Methods

To investigate the variability of the electric fields across subjects, the electric fields of three different electrode montages were simulated in order to target three different predefined regions-of-interest (ROIs) with tTIS. In a first step, we aimed to identify electrode montages that maximize the electric field inside the defined ROI, and simultaneously, minimize the electric field outside the ROI. Since efficient optimization algorithms are currently not available for tTIS, we adapted optimized electrode positions from the literature in order to target our ROIs with tTIS. The different electrode montages were simulated for different subjects and the resulting electric fields were compared. Additionally, the electric field simulations for tTIS were compared to the electric field simulations for conventional tACS, targeting the same ROIs, with optimized electrode positions for both stimulation techniques^26^. Since we expect physiological effects in the gray matter of the brain, we restricted our analysis to this compartment.

### Participants

To test the variability of the electric fields of tTIS across subjects, we simulated different electrode montages on structural MRI scans of 25 different subjects taken from an existing dataset (structural MRIs taken from Kasten et al.^35^). The data was initially acquired from 40 healthy volunteers (age: 24 ± 3 years), recruited at the Carl von Ossietzky University of Oldenburg and was counterbalanced for sex. All subjects were right-handed, without history of neurological and psychiatric disorder, non-smoker, medication free at the days of recordings and had normal or corrected to normal vision. Participants gave written informed consent. The study by Kasten et al.^35^ was approved by the committee for Research Impact assessment and Ethics of the University of Oldenburg and conducted in accordance with the declaration of Helsinki.

MRI images were acquired using a Siemens Magnetom Prisma 3 T whole-body MRI scanner (Siemens, Erlangen, Germany). A T1-weighted 3-D sequence (MPRAGE, TR = 2000 ms, TE = 2.07 ms) with a slice thickness of 0.75 mm was used.

### Electric field simulations

Electric field simulations were performed with MATLAB R2018b and the SimNIBS 3.0 toolbox^36^. A tetrahedral head mesh was created from the MRIs (for detailed information see “Participants”) using SimNIBS’s *headreco* that integrates SPM12 and CAT12 for segmentation with meshfix and gmsh for meshing. After visual inspection of the segmentation results (specifically, we focused on the skull segmentation), we excluded 15 subjects due to obvious segmentation errors, resulting in 25 subjects (age: 25 ± 3 years, 12 females, 13 males). Default conductivities of the toolbox were used for the different compartments (0.126 S/m for white matter, 0.275 S/m for gray matter, 1.654 S/m for csf, 0.01 S/m for bone, 0.465 S/m for skin)^36^. The values of the conductivities represent average values from several references (e.g. see Table 1 in Wagner et al.^37^). They fall well within the range of conductivities observed in measurements from fresh or live tissues near body temperature for frequencies ranging from 0 to 100 kHz^38^. For the finite-element methods (FEM)-based electric field calculations, rubber stimulation electrodes with a diameter of 1 cm and conductivity of 29.4 S/m were placed at known 10–20 EEG system electrode sites. The electrically conductive, adhesive paste (conductivity of 1 S/m) underneath the electrodes were estimated with a thickness of 2 mm. SimNIBS 3.0 was used to simulate the electrical fields for each electrode pair separately. In order to investigate the distribution of the total electric field generated from the temporal interfering fields of two electrode pairs, further calculation steps were necessary. The spatial distribution of the envelope modulation amplitude was computed with the formula used by Grossmann et al.^16^

$$\left|{\overrightarrow{E}}_{AM}\left(\overrightarrow{n},\overrightarrow{r}\right)\right|= \left|\left|\left({\overrightarrow{E}}_{1}\left(\overrightarrow{r}\right)+{\overrightarrow{E}}_{2}\left(\overrightarrow{r}\right)\right)\cdot \overrightarrow{n}\right|- \left|\left({\overrightarrow{E}}_{1}\left(\overrightarrow{r}\right)-{\overrightarrow{E}}_{2}\left(\overrightarrow{r}\right)\right)\cdot \overrightarrow{n}\right|\right|$$

where E1(r) and E2(r) are the two electrical fields generated for the first and second electrode pairs at location r(x,y,z) r(x,y,z), $\left|{\overrightarrow{E}}_{1}\left(\overrightarrow{r}\right)\right|$ and $\left|{\overrightarrow{E}}_{2}\left(\overrightarrow{r}\right)\right|$ are amplitude of ${\overrightarrow{E}}_{1}\left(\overrightarrow{r}\right)$ and ${\overrightarrow{E}}_{1}\left(\overrightarrow{r}\right)$ respectively at a position $\overrightarrow{r}$; and $\overrightarrow{n}$ is a unit vector along the direction of interest, thus $\left|{\overrightarrow{E}}_{AM}\left(\overrightarrow{n},\overrightarrow{r}\right)\right|$ is a projection of the modulation amplitude in a direction tangential to the unit vector. The maximal envelope modulation amplitude of this signal was calculated using

$$\left| {\vec{E}_{{AM}}^{{max}} \left( {\vec{r}} \right)} \right| = \left\{ {\begin{array}{*{20}l} {2\left| {\vec{E}_{2} \left( {\vec{r}} \right)} \right|} \hfill & {if\;\left| {\vec{E}_{2} \left( {\vec{r}} \right)} \right| < \left| {\vec{E}_{1} \left( {\vec{r}} \right)} \right|\;{\text{cos}}\alpha } \hfill \\ {{{2\left| {\vec{E}_{2} \left( {\vec{r}} \right) \times \left( {\vec{E}_{1} \left( {\vec{r}} \right) - \vec{E}_{2} \left( {\vec{r}} \right)} \right)} \right|} \mathord{\left/ {\vphantom {{2\left| {\vec{E}_{2} \left( {\vec{r}} \right) \times \left( {\vec{E}_{1} \left( {\vec{r}} \right) - \vec{E}_{2} \left( {\vec{r}} \right)} \right)} \right|} {\left| {\vec{E}_{1} \left( {\vec{r}} \right) - \vec{E}_{2} \left( {\vec{r}} \right)} \right|}}} \right. \kern-\nulldelimiterspace} {\left| {\vec{E}_{1} \left( {\vec{r}} \right) - \vec{E}_{2} \left( {\vec{r}} \right)} \right|}}} \hfill & {otherwise} \hfill \\ \end{array} } \right.$$

The equations were adapted from Grossman et al. ^16^ and Rampersad et al.^26^ and implemented in a MATLAB function (function is implemented in the SimNIBS toolbox).

### Simulation of the electric field during tTIS on the MNI brain

Several studies use the well-known Montreal Neurological Institute (MNI) average brain^39^ (brain template, that is created from 3D brain MRI images of 152 normal subjects) in order to predict the electric field distribution and strength of different brain stimulation techniques. Therefore, we simulated the electric field of different electrode montages targeting different ROIs on the MNI brain in order to investigate the spatial distribution and the strength of the electric field due to different electrode montages stimulating predefined ROIs with tTIS. The ROIs were defined as the left hippocampus, the left motor cortex and the thalamus (see Fig. 1b for the positions and sizes of the ROIs). In order to target the left hippocampus and the left motor cortex, we used the optimized electrode montages developed by Rampersad et al.^26^. The authors showed that the optimal electrode positions for tTIS to target the left hippocampus are AFz/P7, and AF4/P9 (see Fig. 1c) and for the left motor cortex F1/CP1, and FC1/CP3 (see Fig. 1c). These electrode montages lead to the maximal electric field strength over all directions (E_x_, E_y_, E_z_) in the ROI. The authors also showed other electrode montages considering that neurons respond preferentially to stimulation when the field is oriented along the predominant direction of the neuron. However, the current study simulates the electrode montages that maximize the electric field strength in a given location over all directions. In addition, we aimed to simulate the electric field in order to target the thalamus. To this end, we simulated different electrode montages (we limited the electrode positions to frontal and parietal/occipital electrode positions) and chose the electrode montage that maximizes the electric field inside the thalamus while minimizing the electric field outside the thalamus by visual inspection. We end up with the electrode positions F7/PO7, and F8/PO8 (see Fig. 1c). We used the same electrode positions for tACS in order to target the thalamus. For all electric field simulations, the current strength was defined at 1 mA per electrode pair.

In order to characterize the electric field distribution and strength, we calculated the mean electric field inside the ROI’s, as well as the maximum of the electric field inside vs. outside the ROI by using the AAL-atlas^40^. Non-invasive electric brain stimulation with a current strength of 1 mA leads typically to an electric field of approx. 0.1–0.3 V/m inside the brain^29–31^. Since tTIS is based on similar mechanisms of action as tACS, we aim to achieve comparable electric field strength with tTIS than with well-known and established non-invasive brain stimulation techniques. Based on this knowledge, we characterized the focality of the different simulations: we calculated the proportion of voxel inside the ROI that exposed to field strength greater than 0.1 V/m, and 0.2 V/m. In order to complement our analyses with an index of focality, we also computed the proportion of voxels that exposed to field strength with greater than 0.1 V/m, and 0.2 V/m inside the whole gray matter volume.

### Simulation of the electric field during tTIS on individual brains

To evaluate the variability across subjects, we computed spatial correlations between different electric field simulations of the subjects and the predicted electric field, simulated on the MNI brain. Additionally, we computed spatial correlations across the electric field simulations of the different subjects and calculated characteristics of the electric fields (mean electric field inside the ROI, maximum of the electric field inside/outside the ROI, proportion of voxel that exposed to field strength > 0.1 V/m, and 0.2 V/m in the ROI and for the whole gray mater volume, as explained in “Simulation of the electric field during tTIS on the MNI brain”).

### Comparison of the electric fields during tTIS and conventional tACS

In addition to the optimal electrode montages for tTIS, Rampersad et al.^26^ showed the optimal electrode montages for tACS to target the left hippocampus and the left motor cortex. The maximal total current strength was defined to be 2 mA. For tACS, the optimal current ratio (current ratio between both electrode pairs), to target the left hippocampus is 0.1 (AF3/P7 stimulate with 1.8 mA; AFz/TP7 stimulate with 0.18 mA). In order to target the left motor area with tACS, the optimal current ratio is 10 (F1/CP1 stimulate with 0.18 mA; FC1/CP3 stimulate with 1.8 mA). The adapted electrode montages of tACS are visualized in Fig. 1c. For tTIS, the current ratio for all ROI’s is 1 (resulting in 1 mA per electrode pair). To compare the electric fields of tTIS with conventional tACS, we contrasted the electric field simulations of tTIS with the electric field simulations of tACS for the left hippocampus, the left motor cortex and the thalamus. We compared the spatial distribution and electric field strength of both brain stimulation techniques by contrasting the characteristics of the electric field of tTIS and tACS (mean electric field inside the ROI, maximum of the electric field inside/outside the ROI, proportion of voxel that exposed to field strength greater than 0.1 V/m and 0.2 V/m inside the ROI and for the whole gray mater volume).

## Results

For the analysis, we investigated the amplitude of the low frequency envelope of tTIS (calculated as explained in “Electric field simulations”), in contrast to the amplitude of tACS. Note that the electric fields of the tTIS montages have a different distribution and strength for the total electric field itself, resulting from both electrode pairs oscillating at slightly different high frequencies. However, the focus of the current study is to compare the electric field of the envelope frequency of tTIS across subjects, and against tACS.

### ROI: left hippocampus

**Figure 2:**
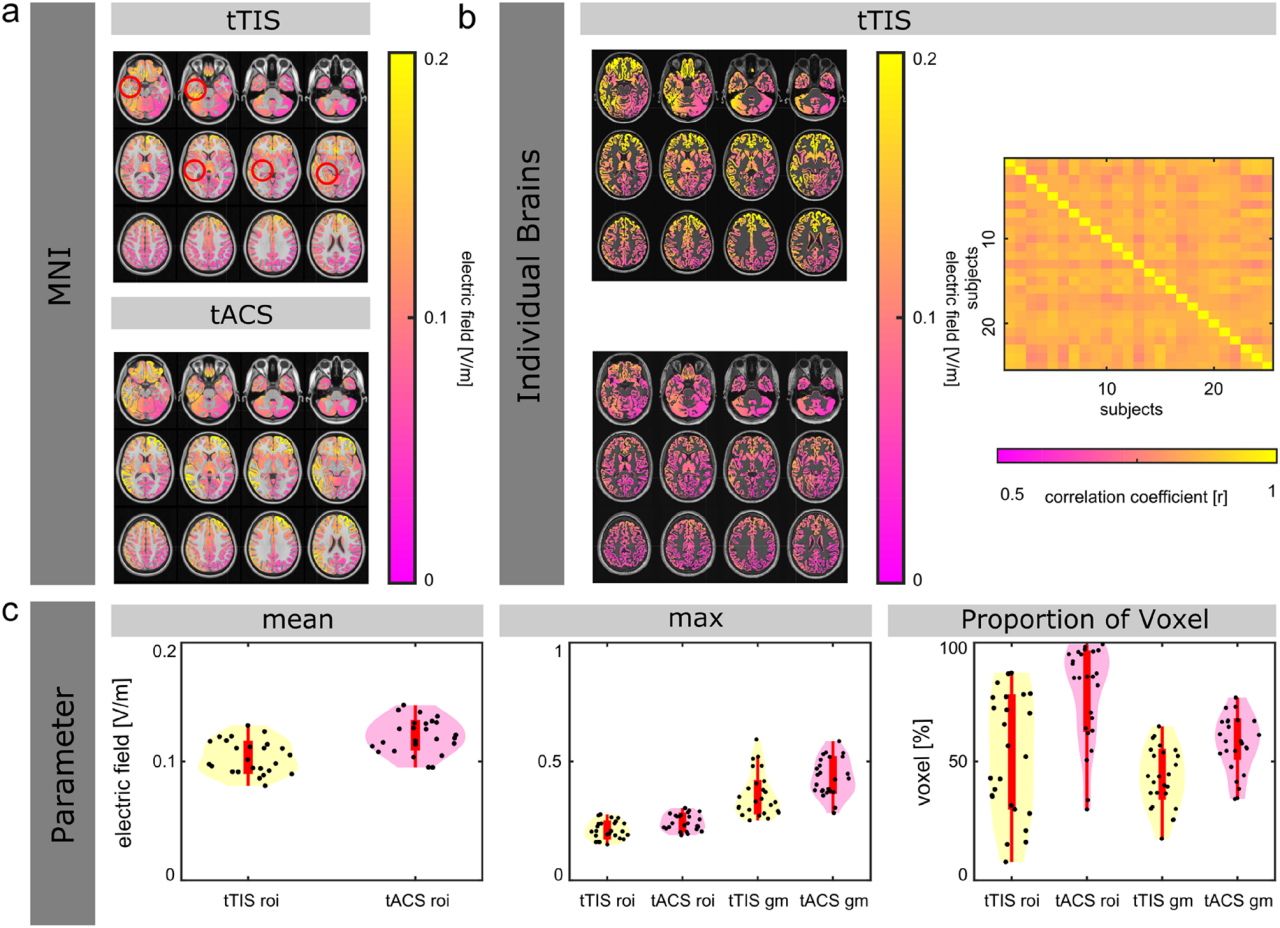
Electric field simulation results for transcranial temporal interference stimulation (tTIS) and transcranial alternating current stimulation (tACS), targeting the left hippocampus. (**a**) Electric field distribution on the MNI brain for tTIS and tACS. (**b**) Electric field distribution for two representative subjects targeting the left hippocampus for tTIS (left). Spatial correlation of the electric fields over the whole brain across all subjects (right). (**c**) Parameter that characterize the electric field simulations for tTIS and tACS (mean electric field inside the ROIs—bottom left, the maximum of the electric fields inside vs. outside the ROIs—bottom middle, proportion of voxel that exposed to field strength greater than 0.1 V/m inside the ROIs and for the whole gray matter—bottom right). Violin plots indicate the distribution of the underlying data. Note that the violin plots include a boxplot (in the center of the violin with the median (white dots), 25% quartile and 75% quartile, upper- and lower adjacent value. Additionally, individual data points are overlayed (black dots).

The electric fields for both brain stimulation methods, tTIS and tACS, targeting the left hippocampus on the MNI brain are visualized in Fig. 2a. Overall, tTIS and tACS induce similar mean electric field strength (difference of 0.01 V/m), as well as to similar maxima of the electric field inside the left Hippocampus (difference of 0.03 V/m). However, tACS results in a higher maximum of the electric field outside the left hippocampus (difference of 0.1 V/m). For tACS, approx. 15% more of the voxels inside the left hippocampus, as well as for the whole gray matter volume, are exposed to electric field strengths of greater than 0.1 V/m (see Table 1, left Hippocampus).

**Table 1:** MNI brain. Summary of the parameter that characterize the electric field distribution on the MNI brain for the left hippocampus, the left motor area and the thalamus: The mean electric field inside the ROI (mean e ROI), the maximum of the electric field inside vs. outside the ROI (max e ROI/max e gm), and the proportion of voxel that exposed to field strength greater than 0.1 V/m inside the ROI and for the whole gray matter (ROI > 0.1 V/m/Gm > 0.1 V/m).

	Left hippocampus		Left motor area		Thalamus	
	tTIS	tACS	tTIS	tACS	tTIS	tACS
Mean e ROI (in V/m)	0.11	0.12	0.1	0.12	0.09	0.09
Max e ROI (in V/m)	0.17	0.2	0.17	0.21	0.18	0.19
Max e gm (in V/m)	0.28	0.37	0.26	0.28	0.27	0.31
ROI > 0.1 V/m (in %)	61.44	77.33	30	78.7	21.01	32.15
ROI > 0.2 V/m (in %)	0	0	0	0.3	0	0
Gm > 0.1 V/m (in %)	39.3	52.71	5.5	14.3	1.22	32.1
Gm > 0.2 V/m (in %)	0.04	2.29	0.13	0.21	0	0.2

For tTIS, the electric field simulations targeting the left hippocampus are visualized for two representative subjects in Fig. 2b. The electric fields of the individual brains correlate with a mean of r = 0.85 (std = 0.01, median = 0.84, 75th percentile = 0.86, 25th percentile = 0.83, minimum = 0.83, maximum = 0.87, outliers = 0) with the predicted electric field on the MNI brain. The averaged correlation between individual simulation results in r = 0.85 (std = 0.01, median = 0.85, 75th percentile = 0.86, 25th percentile = 0.82, minimum = 0.77, maximum = 0.9, outliers = 2). The individual correlation coefficients are displayed in Fig. 2b.

The values for single subjects (mean electric fields inside left hippocampus and maxima of electric fields inside/outside left hippocampus, proportion of voxel that exposed to field strength greater than 0.1 V/m inside the ROI, as well as for the whole gray matter volume) are visualized in Fig. 2c. Overall, similar patterns are shown for the individual brains as for the MNI brain. However, while also in the same direction, the difference of voxels that expose to field strength greater than 0.1 V/m inside the left hippocampus is approx. 25% between tTIS and tACS (see Table 2, left hippocampus).

**Table 2:** Individual brains. Summary of the parameter that characterize the electric field distribution across subjects for the left hippocampus, the left motor area and the thalamus: The mean electric field for all subjects inside the ROI (mean e ROI), the maximum of the electric field for all subjects inside vs. outside the ROI (max e ROI/max e gm), and the proportion of voxel that exposed to field strength greater than 0.1 V/m inside the ROI and for the whole gray matter (ROI > 0.1 V/m/Gm > 0.1 V/m). Standard deviations are displayed in brackets.

	Left hippocampus		Left motor area		Thalamus	
	tTIS	tACS	tTIS	tACS	tTIS	tACS
Mean e ROI (in V/m)	0.1 (0.01)	0.1 (0.01)	0.13 (0.02)	0.14 (0.02)	0.08 (0.01)	0.09 (0.01)
Max e ROI (in V/m)	0.21 (0.04)	0.24 (0.03)	0.25 (0.04)	0.25 (0.04)	0.2 (0.03)	0.21 (0.03)
Max e gm (in V/m)	0.36 (0.09)	0.43 (0.08)	0.36 (0.08)	0.43 (0.09)	0.27 (0.04)	0.36 (0.08)
ROI > 0.1 V/m (in %)	52.04 (25.33)	78.57 (20.34)	81.42 (22.2)	85.64 (19.84)	10.77 (13.38)	24.93 (27.16)
ROI > 0.2 V/m (in %)	0.43 (0.65)	1.57 (1.75)	5.19 (6.19)	7.13 (8.91)	0.03 (0.05)	0.04 (0.06)
Gm > 0.1 V/m (in %)	43.26 (12.51)	57.35 (11.87)	15.11 (5.52)	17.52 (5.69)	1.68 (1.99)	39.53 (18.62)
Gm > 0.2 V/m (in %)	1.44 (1.59)	6.2 (4.24)	1.23 (0.91)	2.43 (1.56)	0 (0)	0.79 (0.67)

### ROI: left motor area

**Figure 3:**
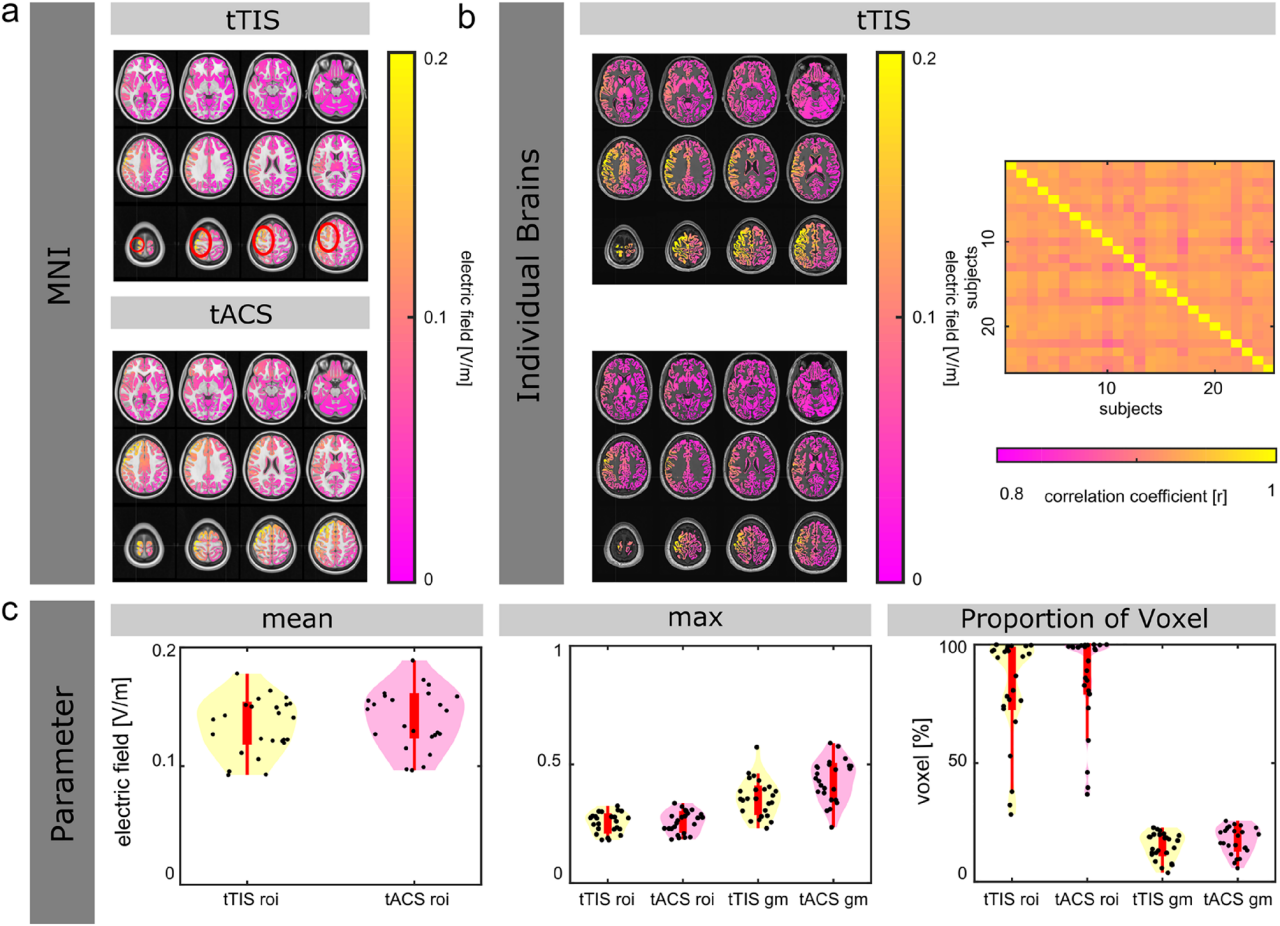
Electric field simulation results for transcranial temporal interference stimulation (tTIS) and transcranial alternating current stimulation (tACS), targeting the left motor area. (**a**) Electric field distribution on the MNI brain for tTIS and tACS. (**b**) Electric field distribution for two representative subjects targeting the left motor area for tTIS (left). Spatial correlation of the electric fields over the whole brain across all subjects (right). (**c**) Parameters that characterize the electric field simulations for tTIS and tACS (mean electric field inside the ROIs—bottom left, the maximum of the electric fields inside vs. outside the ROIs—bottom middle, proportion of voxel that exposed to field strength greater than 0.1 V/m inside the ROIs and for the whole gray matter—bottom right).

The electric fields for both stimulation methods, tTIS and tACS, targeting the left motor area on the MNI brain are visualized in Fig. 3a. Overall, the results are in line with the results for the left hippocampus: similar mean- and maxima of the electric fields inside the left motor area, as well as higher percentage of voxels that expose to field strength greater than 0.1 V/m inside the left motor area, and for the whole gray matter volume for tACS. However, while also in the same direction, the difference of voxels that expose to field strength greater than 0.1 V/m inside the left motor area is nearly 50% between tTIS and tACS. The maximum of the electric field inside the left motor area is similar for both tTIS and tACS (difference of 0.02 V/m).

For tTIS, the electric field simulations targeting the left motor area are visualized for two representative subjects in Fig. 3b. The electric fields of the individual brains correlate with a mean of r = 0.94 (std = 0.01, median = 0.94, 75th percentile = 0.94, 25th percentile = 0.93, minimum = 0.92, maximum = 0.95, outliers = 0) with the predicted electric field on the MNI brain. The mean correlation across all subjects is r = 0.93 (std = 0.002, median = 0.92, 75th percentile = 0.93, 25th percentile = 0.92, minimum = 0.88, maximum = 0.94, outliers = 6). The individual correlation coefficients are displayed in Fig. 3b.

The values for single subjects (mean electric fields inside left motor area and maxima of electric fields inside/outside left motor area, proportion of voxel that exposed to field strength greater than 0.1 V/m inside the ROI, as well as for the whole gray matter) are visualized in Fig. 3c.

Overall, similar patterns are shown for both ROIs (left hippocampus and left motor area) regarding the parameter characterizing the electric field distribution and strength, when comparing tTIS with tACS: tACS resulted in only slightly stronger, or the same mean electric fields inside ROIs compared to tTIS, and only slightly higher, or the same maximal electric field inside the ROIs. However, tACS led to higher maximal electric field outside the ROI (see Table 2, left motor area).

### ROI: thalamus

**Figure 4:**
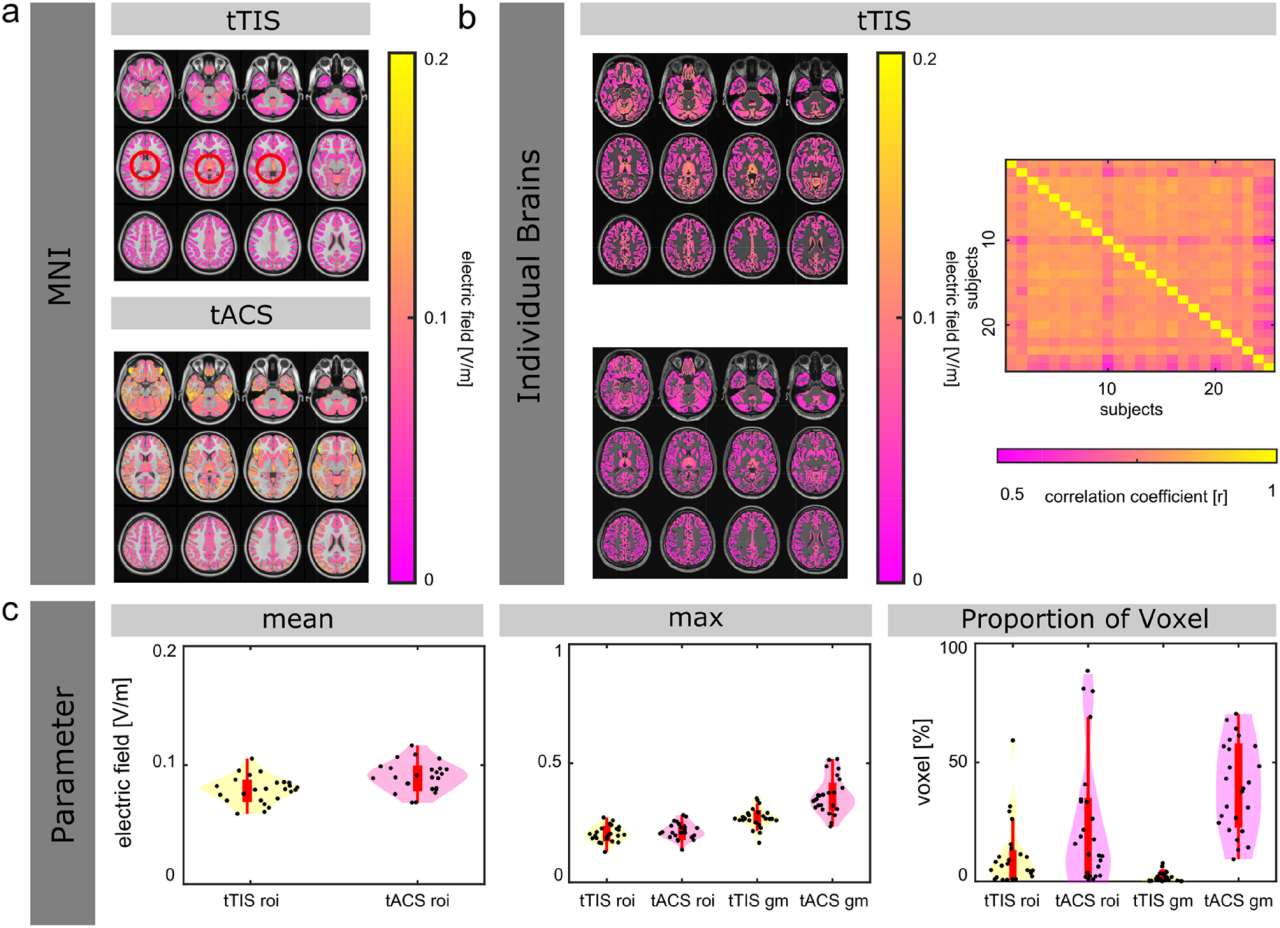
Electric field simulation results for transcranial temporal interference stimulation (tTIS) and transcranial alternating current stimulation (tACS), targeting the thalamus. (**a**) Electric field distribution on the MNI brain for tTIS and tACS. (**b**) Electric field distribution for two representative subjects targeting thalamus for tTIS (left). Spatial correlation of the electric fields over the whole brain across all subjects (right). (**c**) Parameter that characterize the electric field simulations for tTIS and tACS (mean electric field inside the ROIs—bottom left, the maximum of the electric fields inside vs. outside the ROIs—bottom middle, proportion of voxel that exposed to field strength greater than 0.1 V/m inside the ROIs and for the whole gray matter—bottom right).

The electric fields for both stimulation methods, tTIS and tACS, targeting thalamus on the MNI brain are visualized in Fig. 4a. Overall, the results are in line with the patterns shown for the left hippocampus and the left motor area. However, only 1.22% of the voxel of the whole gray matter volume expose to a field strength greater than 0.1 V/m for tTIS. In contrast, 32.1% of the voxel of the whole gray matter volume expose to a field strength greater than 0.1 V/m for tACS (see Table 1, thalamus).

The electric field simulations targeting the thalamus are visualized for two representative subjects in Fig. 4b. For tTIS, the electric fields of the individual brains correlate with a mean of r = 0.77 (std = 0.03, median = 0.78, 75th percentile = 0.79, 25th percentile = 0.77, minimum = 0.67, maximum = 0.82, outliers = 2) with the predicted electric field of the MNI brain. The mean correlation across all subjects is r = 0.79 (std = 0.01, median = 0.79, 75th percentile = 0.81, 25th percentile = 0.76, minimum = 0.57, maximum = 0.84, outliers = 13). The individual correlation coefficients are displayed in Fig. 4b.

The values for single subjects are visualized in Fig. 4c (mean electric fields inside thalamus and maxima of electric fields inside/outside thalamus, proportion of voxel that exposed to field strength greater than 0.1 V/m inside the ROI, as well as for the whole gray matter).

Overall, similar patterns are shown for all ROIs (left hippocampus, left motor area and thalamus) regarding the parameter characterizing the electric field distribution and strength. However, similar to the results on the MNI brain for the thalamus: only 1.68% (std = 1.99) of the voxel of the whole gray matter volume expose to a field strength greater than 0.1 V/m for tTIS. In contrast, 39.53% (std = 18.62) of the voxel of the whole gray matter volume expose to a field strength greater than 0.1 V/m for tACS (see Table 2, thalamus).

## Discussion

The variability of electric fields resulting from stimulation using the relatively new brain stimulation technique tTIS across different subjects has hitherto not been extensively studied. It is important, however, to investigate how the electric fields differ across subjects with the same electrode montage^26^ in order to account for plausible differences regarding the stimulation effect. Differences regarding the electric field could lead to different stimulation effects^35^. Therefore, the aim of the current study was to investigate the variability of electric fields due to tTIS across 25 subjects. Moreover, we compared the electric fields of tTIS with the electric fields of conventional tACS by targeting different ROI’s with both brain stimulation technique. In this study we found that the electric fields generated by tTIS have variable strengths on single subject level inside the ROIs (left hippocampus, left motor area and thalamus). In addition, when compared to tACS, tTIS stimulates more focally meaning that it induces much weaker co-stimulation of cortical areas close to the stimulation electrodes. Electric fields generated inside the ROI were, however, comparable for both methods.

Overall, electric fields during tTIS targeting different ROIs (left hippocampus, left motor area and thalamus) correlate highly across all subjects irrespective of the target ROI (averaged correlation coefficient across subjects ranging from 0.79 to 0.93). However, the individual correlation coefficients range from 0.57 to 0.94, indicating variability of the electric field distribution across individuals. A similar result can be seen when considering the strength of the electric field. The mean electric field across subjects ranged from 0.08 V/m to more than 0.13 V/m inside the different ROIs. On single subject level, the strength of the electric fields are highly variable, especially the proportion of voxels showing more than 0.1 V/m. For several subjects, more than 80% of the voxels inside the different ROIs are stimulated over 0.1 V/m, whereas in other subjects less than 10% of the voxels inside the ROIs exposed to field strength stronger than 0.1 V/m. The variability of the results indicates that a precise and individual electrode montage is necessary to achieve similar electric field strengths inside the target area across subjects.

It was shown that multi-channel tTIS increase the focality while reducing scalp sensations in computational modeling- and animal-experiments^41^. Therefore, further research to develop optimization algorithms (addressing single-channel and multi-channel tTIS strategies) could be promising in order to individually adjust the electrode montages and ensure consistent stimulation of relatively small deep brain targets. However, presumably due to the high conductivity of CSF, targeting deep brain areas precisely, without stimulating brain regions outside the ROI will likely remain challenging.

Interindividual variability of electric fields have been investigated for established non-invasive electric brain stimulation techniques like tACS/tDCS in previous studies. Possible anatomical characteristics that affect the electric field distribution, and especially the focality, differently across subjects might be the thickness of cerebrospinal fluid^42^. In addition, higher head-, skin-, and skull-volume seems to be associated with lower electric field strength inside the brain^43^. Since tTIS is based on similar mechanisms of action as conventional non-invasive electric brain stimulation techniques, similar anatomical characteristics are likely to be responsible for interindividual differences as well.

By comparing the electric fields of tTIS with the electric fields of conventional tACS, we showed that tTIS is more focal by substantially reducing co-stimulation in cortical areas in the proximity of stimulation electrodes (e.g. see electric field distributions for tTIS and tACS on the MNI brain in Fig. 2a). Across all subjects, electric field simulations for tACS resulted in only slightly stronger mean electric fields inside ROIs as compared to tTIS and, additionally, only slightly higher maximal electric field inside the ROIs. However, tACS led to higher maximal electric fields outside the ROI, especially noticeable for deep brain structures like the left hippocampus montage (on average 0.43 V/m, std = 0.08 for tACS, and 0.36 V/m, std = 0.09 for tTIS) and the thalamus (on average 0.36 V/m, std = 0.08 for tACS, and 0.27 V/m, std = 0.04 for tTIS). Overall, tTIS is more focal (i.e., having much weaker stimulation outside the ROIs) as compared to tACS, when using the optimization strategy put forward by Rampersad et al.^26^. Due to the development of different systematic optimization approaches, especially the tACS electrode montages could probably be optimized more effectively. While tTIS and tACS have comparable electric fields inside the ROI (mean/max inside ROI), the maximal electric field outside the ROI is higher for tACS. In addition, the proportion of voxels of the whole gray matter volume (for deep brain structures, such as the hippocampus and the thalamus) is higher for tACS, especially noticeable for the thalamus. The direct comparison of the electric fields of tTIS and tACS are, however, challenging. Whereas the tACS stimulation waveform is close to a perfect sine wave (e.g. stimulation with 10 Hz), the envelope of the amplitude modulated signal of tTIS is not perfectly sinusoidal (Fig. 1a, right panel). Moreover, the electric field distribution of the envelope frequency of tTIS is smaller than the total electric field itself, resulting from both electrode pairs oscillating at slightly different high frequencies. Therefore, the influence of the non-sinusoidal signal, as well as the influence of the high frequencies has to be investigated in humans.

Further research should also investigate the effects of a simultaneous stimulation of non-target cortical areas. Namely, there may be some clinical situations, where it might be especially important to stimulate a deep brain area as strongly as possible, while the specificity (i.e. the stimulation of additional cortical areas) is less problematic. With respect to research questions, the co-stimulation of tACS seems to be more problematic since the effects of stimulation cannot be ascribed solely to the stimulation of the deep brain structure^44^. However, control stimulation conditions targeting only the co-stimulated cortical areas might help to investigate this question.

The results of the current study indicate that the maxima of the electric fields outside the ROIs are slightly higher than inside the ROIs, even when stimulating with tTIS (however, the difference is much lower compared to tACS). The use of optimization algorithms might address this issue in further studies. One major limitation of the current study is, that we did not use an optimization algorithm in order to target the defined ROIs. There might be electrode positions that target the ROI more precisely on the MNI brain. The used electrode montages by Rampersad et al.^26^ are optimized for one single subject. Therefore, it would be especially promising for further research to develop an optimization algorithm to maximize the electric field inside the ROI, while minimizing the electrical field outside the ROI using tTIS. Additionally, since only T1 MRI scans were used for the current study, the accuracy of the skull segmentation could be improved, e.g. with an additional T2 MRI scan. However, we ruled out by visual inspection that low correlations with mean fields, as seen in some of the subjects, were cause by obvious segmentation errors. Moreover, since stimulation with high frequencies leads to less somatosensory sensations, a higher current could be used in order to target deep brain areas with tTIS. Lee et al.^24^ employed realistic finite head models for the optimization of tTIS. They suggested that customized tTIS based on numerical field analysis is expected to enhance the overall effectiveness of noninvasive deep brain stimulation.

It has to be noted that the robustness of the results of the current study is linked to the electric field threshold choice of 0.1 V/m resulting from a stimulation intensity of 1 mA per electrode pair. Based on literature^29–31^, we used different thresholds in order to characterize the electric field distribution and strength. As there is no common agreement which stimulation intensities are required for tACS and tTIS to modulate brain activity, this approach describes the general coverage of the electric fields due to the different electric brain stimulation techniques. Therefore, we calculated the proportions of voxels that expose to field strength greater than 0.1 V/m, and 0.2 V/m. However, further research is needed to investigate this concern more precisely. Obviously, given the linear dependence of the electric field on the stimulation current, identical results for focality would be obtained for a threshold of 0.2 V/m, and 0.3 V/m when doubling the stimulation currents to 2 mA per electrode pair, which is still in a practically feasible range. Moreover, the electric fields were simulated for electrode montages that maximize the electric field strength in given locations (three different ROIs) over all directions (E_x_, E_y_, E_z_). This does not consider that neurons respond preferentially to stimulation when the field is oriented along the predominant direction of the neuron. Overall, further research is needed to investigate precise parameters of the electric field that correlate with stimulation effects (e.g. electric field strength, direction of electric field).

Also the ability of transcranial magnetic stimulation (TMS) to reach deep brain structures has been tested and specific TMS coils have been made available that maximize the electric field intensities^45^. However, also TMS of deeper structures suffers from a low focality and a substantial co-stimulation of more superficial structures^46^. tTIS addresses this issue by stimulating deep brain structures while reducing stimulation of the overlaying cortex. However, as shown in this study, individual electrode montages are necessary. In addition, the mechanisms of action differ between TMS (capable of supra-threshold stimulation) and tTIS (sub-threshold stimulation), suggesting that they might have partly complementary application profiles.

Since current tTIS studies are limited to electric field simulations, animal studies, or computational models^16,19–21,23,26–28^, it is still unknown whether and how tTIS leads to stimulation effects in humans. More specifically, it is unclear whether tTIS works in humans similar to the experiments in mice^16,41^, due to the fundamental differences between mice and human regarding the size and anatomy of the brain. To this end, further research is essential in order to investigate the stimulation effects of tTIS in the human brain.

## Data Availability

The datasets analyzed during the current study are available from the corresponding author on reasonable request.
